# Bisphenol A in Thermal Paper Receipts: Taylor et al. Respond

**DOI:** 10.1289/ehp.1104004R

**Published:** 2012-01-01

**Authors:** Julia A. Taylor, Frederick S. vom Saal, Wade V. Welshons, Bertram Drury, George Rottinghaus, Patricia A. Hunt, Pierre-Louis Toutain, Céline M. Laffont

**Affiliations:** University of Missouri, Columbia, Missouri, E-mail: TaylorJA@Missouri.edu; School of Molecular Biosciences, Washington State University, Pullman, Washington; INRA, TOXALIM (Research Centre in Food Toxicology) and Ecole Nationale Veterinaire de Toulouse, Université de Toulouse, Toulouse, France

We agree with Schwartz and Landrigan that there is a need for change in the regulatory system for chemicals used in products in the United States. Bisphenol A (BPA) is one of thousands of chemicals of concern, but it provides a striking example of what happens when there is no requirement for premarket testing. Full estrogenic activity was demonstrated for BPA when it was tested for use as a pharmaceutical drug in 1936, which should have precluded its use in the wide range of products that results in continuous exposure (Stahlhut et al. 2009). The findings we reported in our article (Taylor et al. 2011) show that clearance of BPA in mice, monkeys, and humans does not differ, and years of research has demonstrated that mice and rats are valid models for predicting the long-term adverse consequences of developmental exposure to estrogenic chemicals. A vast and rapidly growing number of studies with experimental animals (Richter et al. 2007) and humans (Braun and Hauser 2011) report adverse effects later in life as a result of exposure to BPA during development.

In the 2003–2004 National Health and Nutrition Examination Survey (NHANES) study, the Centers for Disease Control and Prevention estimated that 93% of people in the United States are exposed to BPA, with higher exposures in children than adults. The potential exposure of fetuses and infants to BPA is especially concerning because BPA is not metabolized effectively during these highly sensitive stages of human development. Our data (Taylor et al. 2011) indicate that to reach the median serum levels of unconjugated (bioactive) BPA reported in multiple biomonitoring studies (Vandenberg et al. 2010), exposure must be far higher than predicted by the Food and Drug Administration (FDA) based on its risk assessment of BPA (FDA 2008); these government estimates (FDA 2008) are based on kinetics after acute oral exposure and the assumption that food and beverage packaging is the only source of BPA exposure. However, data from the 2003–2004 NHANES (Stahlhut et al. 2009) confirmed that BPA exposure is likely to be from multiple sources—including thermal receipt paper—and there is evidence that in adults different forms of exposure do not have the same metabolic profile (Sieli et al. 2011).

We find it disturbing that government agencies continue to argue that the public should not be concerned about BPA because daily exposures are below “safe” levels. This conclusion is based on flawed studies using outdated approaches. We agree with Schwartz and Landrigan that we have to stop repeating the same mistakes made previously with chemicals such as lead, for which, after decades of repeatedly lowering “safe” exposure estimates, the current predicted “safe” level is still above levels now known to cause adverse effects. For endocrine-disrupting chemicals there are no threshold doses below which exposures are safe (Sheehan 2006), a reality that regulators are unwilling to acknowledge.
